# Supplementary material for: Extracts of Fruits and Plants Cultivated In Vitro of Aristotelia chilensis (Mol.) Stuntz Show Inhibitory Activity of Aldose Reductase and Pancreatic Alpha-Amylase Enzymes
Source: Plants (Basel). 2022 Oct 20;11(20):2772. doi: 10.3390/plants11202772 (PMC9610771; doi:10.3390/plants11202772)
Supplement: Supplementary file 1 [file plants-11-02772-s001.zip › plants-1934296-supplementary.pdf]

plants

MDPI

5. Supplementary material

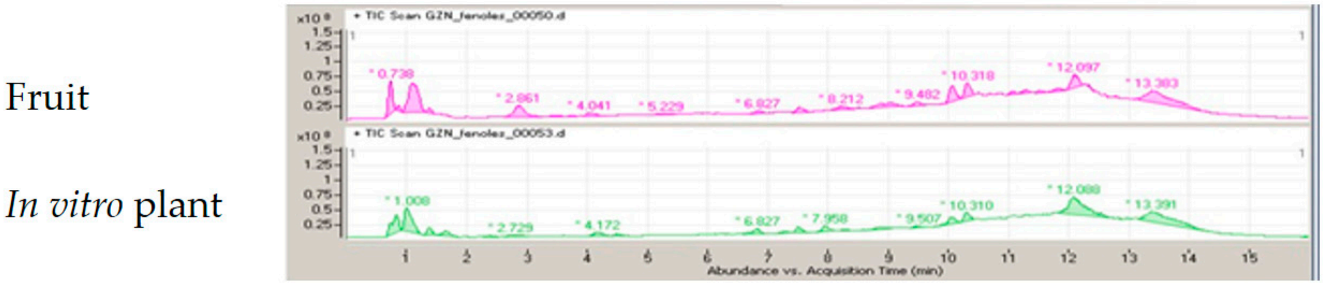

Figure S1. LC-MS/MS chromatogram in positive ionization mode for aqueous extracts of *A. chilensis*. Minutes 1 to 15.

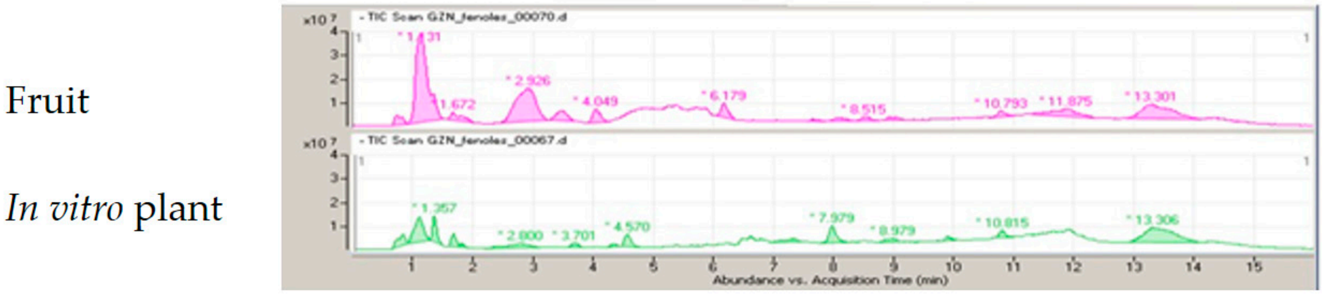

Figure S2. LC-MS/MS chromatogram in negative ionization mode for aqueous extracts of *A. chilensis*. Minutes 1 to 15.
